# Chronic Low-Level Vagus Nerve Stimulation Improves Long-Term Survival in Salt-Sensitive Hypertensive Rats

**DOI:** 10.3389/fphys.2019.00025

**Published:** 2019-01-31

**Authors:** Elizabeth M. Annoni, Dusty Van Helden, Yugene Guo, Brett Levac, Imad Libbus, Bruce H. KenKnight, John W. Osborn, Elena G. Tolkacheva

**Affiliations:** ^1^Department of Biomedical Engineering, University of Minnesota, Minneapolis, MN, United States; ^2^Department of Integrative Biology and Physiology, University of Minnesota, Minneapolis, MN, United States; ^3^Department of Biology, University of Minnesota, Minneapolis, MN, United States; ^4^Department of Electrical Engineering, University of Minnesota, Minneapolis, MN, United States; ^5^LivaNova PLC, Houston, TX, United States

**Keywords:** vagus nerve stimulation, hypertension, survival, autonomic, heart, rat

## Abstract

Chronic hypertension (HTN) affects more than 1 billion people worldwide, and is associated with an increased risk of cardiovascular disease. Despite decades of promising research, effective treatment of HTN remains challenging. This work investigates vagus nerve stimulation (VNS) as a novel, device-based therapy for HTN treatment, and specifically evaluates its effects on long-term survival and HTN-associated adverse effects. HTN was induced in Dahl salt-sensitive rats using a high-salt diet, and the rats were randomly divided into two groups: VNS (*n* = 9) and Sham (*n* = 8), which were implanted with functional or non-functional VNS stimulators, respectively. Acute and chronic effects of VNS therapy were evaluated through continuous monitoring of blood pressure (BP) and ECG via telemetry devices. Autonomic tone was quantified using heart rate (HR), HR variability (HRV) and baroreflex sensitivity (BRS) analysis. Structural cardiac changes were quantified through gross morphology and histology studies. VNS significantly improved the long-term survival of hypertensive rats, increasing median event-free survival by 78% in comparison to Sham rats. Acutely, VNS improved autonomic balance by significantly increasing HRV during stimulation, which may lead to beneficial chronic effects of VNS therapy. Chronic VNS therapy slowed the progression of HTN through an attenuation of SBP and by preserving HRV. Finally, VNS significantly altered cardiac structure, increasing heart weight, but did not alter the amount of fibrosis in the hypertensive hearts. These results suggest that VNS has the potential to improve outcomes in subjects with severe HTN.

## Introduction

Hypertension (HTN) affects over one billion people worldwide and is the most prominent risk factor for cardiovascular disease (Fields et al., 2004; NCD Risk Factor Collaboration [NCD-RisC], 2017). Cardiovascular complications due to HTN result in over nine million deaths annually (World Health Organization [WHO], 2013). Left uncontrolled, high blood pressure (BP) can lead to cardiac remodeling, including left atrial dilation, left ventricular hypertrophy, and impaired ventricular relaxation, thus increasing the risk of heart failure (Lalande and Johnson, 2008). These changes make the heart more susceptible to cardiac arrhythmias, and ultimately influence the morbidity, mortality, and quality of life for patients with chronic HTN. The need to control the rise in BP while also addressing the negative effects of complex co-morbidities makes the clinical management of HTN challenging.

Currently, the majority of hypertensive patients are treated with antihypertensive drugs to control BP. However, many limitations exist, including resistant HTN, inability to tolerate therapy, and non-compliance with the medication regime (Mancia et al., 2007; Egan et al., 2011). There is a need for novel treatment options for hypertensive patients to control BP while also addressing the adverse cardiac effects of HTN. One possible target of new treatments is the altered sympatho-vagal balance, which has been reported in hypertensive patients as a significant contributor to disease progression (Guyenet, 2006; Mancia and Grassi, 2014). Several new therapies for HTN primarily target the overactive sympathetic nervous system with the goal of restoring balance in the autonomic nervous system. For instance, baroreflex activation therapy indirectly suppresses sympathetic activity through activation of the arterial baroreceptor reflex, and renal denervation is an organ-specific ablation approach to inhibit sympathetic activity to the kidney. However, both these therapies have had variable success in controlling BP in hypertensive patients (Li et al., 2016).

Recently, we proposed to specifically target the parasympathetic nervous system using vagus nerve stimulation (VNS), and evaluated the efficacy of 4 weeks of VNS treatment for HTN and HTN-induced heart disease in a rat model of HTN (Annoni et al., 2015). Our study and others have demonstrated the beneficial effects of chronic VNS in treating HTN, and its associated improvement of endothelial function and cardiac electrophysiological properties, leading to suppression of ventricular arrhythmias (Xie et al., 2014; Huang et al., 2015; Chapleau et al., 2016). Antiarrhythmic effects have also been recently demonstrated by our lab in healthy hearts in the absence of sympathetic overdrive (Lee et al., 2016). Further, acute VNS therapy has also shown a beneficial effect on BP and heart rate (HR) dynamics in hypertensive rats (Plachta et al., 2014, 2016; Annoni and Tolkacheva, 2018). However, to date, there has been limited research evaluating the long-term effects of VNS on BP and associated cardiovascular complications in advanced-stage HTN in rats.

The aim of this study is to investigate the effects of low-level, intermittent chronic VNS therapy on the long-term survival of hypertensive rats and to evaluate the impact of VNS therapy on BP control, HR dynamics, and structural cardiac properties.

## Materials and Methods

All experiments conform to the Guidelines for the Care and Use of Laboratory Animals (NIH publication No. 85–23, revised 1996) and the University of Minnesota guidelines for the care and use of animals. See Supplementary Material for surgical procedure details.

### Experimental Design

**FIGURE 1:**
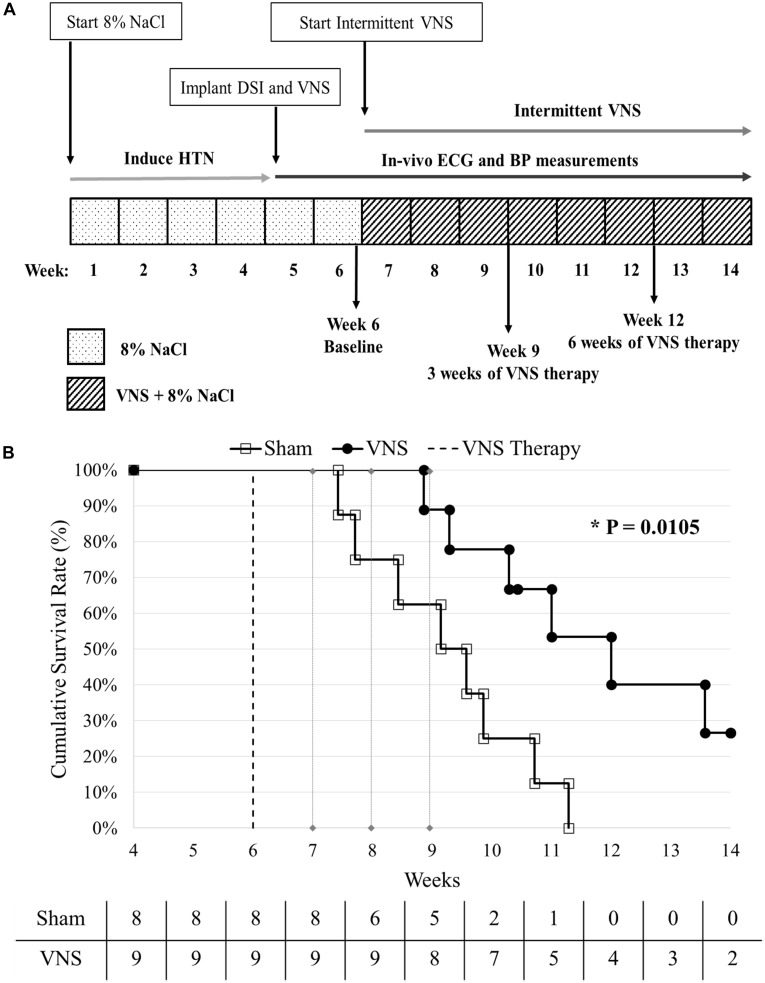
**(A)** Experimental design. HTN was induced using a high salt diet (8% NaCl). At Week 4, the rats were implanted with vagal nerve stimulators and DSI transmitters, and randomly divided into Sham (*n* = 8) and VNS (*n* = 9) groups. VNS therapy was turned on at Week 6 and continuous BP and ECG were recorded until the end of the study. **(B)** Kaplan-Meier event-free survival curves for Sham and VNS rats are statistically different (*P* < 0.05). The dashed vertical line indicates the start of VNS therapy at Week 6. The number of rats remaining in each group is included along the *x*-axis.

Five week old male Dahl salt-sensitive rats (Charles River Laboratories, Wilmington, MA, United States) were fed a high salt (8% NaCl) diet to induce HTN, and this diet was maintained for the duration of the study (see Figure 1A). Four weeks after initiating the high salt diet, the hypertensive rats were implanted with vagus nerve stimulators (Model 103, LivaNova USA, Inc., formerly Cyberonics, Inc., Houston, TX, United States) and physiological telemetry devices (HD S11, DSI, Inc., St. Paul, MN, United States) and randomly divided into two groups: VNS (*n* = 9; functional VNS stimulators) and Sham (*n* = 9; non-functional VNS stimulators). One Sham rat was excluded from analysis due to the delayed recovery from surgery, resulting in *n* = 8 for Sham. At Week 6 (Baseline), the low-level VNS therapy was activated and applied for up to 8 weeks or until an endpoint (death or severe HTN-related events such as a stroke or hematoma) was reached. During VNS therapy, ECG and BP recordings were simultaneously captured through the DSI telemeters for both Sham and VNS rats. The event-free survival rate was calculated with respect to the start of VNS therapy at Week 6. All analyses were performed while blinded to treatment designation. A summary of the data analyses used, the time points selected, and the number of rats in each group can be found in Supplementary Table S1.

### Acute Effects of VNS Therapy

**FIGURE 2:**
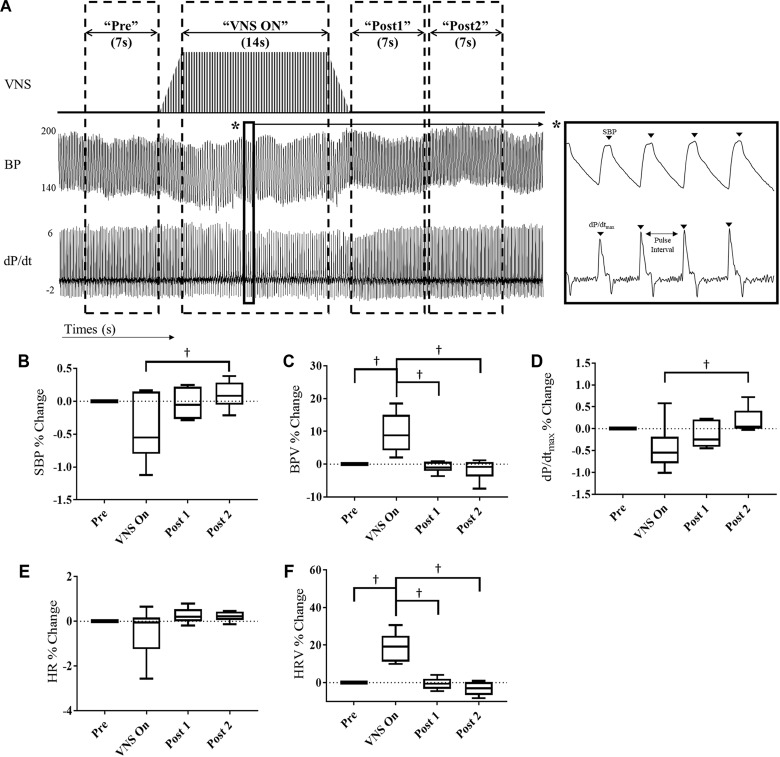
Acute cardiovascular and hemodynamic response of VNS rats (*n* = 6) at Week 9. **(A)** A representative example of VNS stimulation, BP and the BP derivative (dP/dt) traces showing segmentation into “Pre,” “VNS On,” Post 1,” and “Post 2” intervals. Acute SBP **(B)**, BPV **(C)**, contractility (dP/dt_max_) **(D)**, HR **(E)**, and HRV **(F)** responses during VNS therapy in hypertensive rats for “Pre,” “VNS On,” “Post 1,” and “Post 2” intervals shown as percent change with respect to the “Pre” interval. ^†^Indicates statistical significance between intervals.

Acute window analysis was performed as described previously (Annoni and Tolkacheva, 2018). Four-hour data segments were acquired from *Day*_4h_ (10am–2pm) and *Night*_4h_ (10pm–2am) intervals for each VNS rat at 3 and 6 weeks of VNS therapy, Week 9 and Week 12, respectively. Data was segmented into the following windows: “Pre,” “VNS On,” “Post 1,” and “Post 2” as shown in Figure 2A. For each data segment the following values were calculated: systolic blood pressure (SBP), blood pressure variability (BPV), HR, HR variability (HRV), and the contractility, which was determined as the maximum slope of the derivative of the SBP waveform (dP/dt_max_). HRV and BPV were defined as the standard deviation divided by the mean HR and BP values, respectively. The data consists of approximately 400 stimulation episodes per rat that are combined for this analysis. The parameters were calculated for each individual episode of VNS during these intervals and averaged together to reduce variability in measures. Data are presented as percent change with respect to the “Pre” interval values. All data analysis was performed using custom MATLAB code.

### Chronic Effects of VNS Therapy: Disease Progression

Simultaneous ECG and BP data acquired from the DSI telemeters were averaged over 12-h *Day*_12h_ (7am–7pm) and *Night*_12h_ (7pm–7am) intervals, defined by the light-dark cycle of the room. BP and HR measures, including SBP, diastolic blood pressure (DBP), mean arterial pressure (MAP), pulse pressure (PP), HR, and HRV, were calculated.

Baseline BP and HR parameters were quantified as the 3-day average prior to the start of VNS therapy at Week 6. Individual values for *Day*_12h_ and *Night*_12h_ intervals were compared to Baseline data to determine the relative change in BP (ΔSBP), HR (ΔHR), and HRV (ΔHRV) for each animal, as described previously (Annoni et al., 2015). The longitudinal data for Sham and VNS rats were individually fit with a linear regression to quantify the disease progression, and the slopes of the linear fits were averaged for Sham and VNS rats for comparison between groups.

Circadian rhythm analysis was performed at Baseline and at Week 9. A 24-h continuous segment of data was divided into 1-h intervals and fit with a cosinor function using the following equation:

Y=MESOR+Amplitude*cos (Frequency*X+Acrophase),

where *X* is the 1-h average HR or BP data from the Sham or VNS rats, *MESOR* is the mean value of the cosinor function, *Amplitude* is the difference between the peak value and the *MESOR*, and *Acrophase* is the phase shift. The *Frequency* was set to 0.2618 rad/h, corresponding to a 24-h period.

Baroreflex sensitivity (BRS) was quantified using a traditional sequence method for *Day*_4h_ and *Night*_4h_ intervals to compare autonomic tone in the hypertensive rats (Bertinieri et al., 1985).

### Histology and Gross Morphology

After reaching an endpoint of the *in vivo* protocol, excluding death, the hearts were extracted through a midline incision and immediately submerged in cold cardioplegic solution. The hearts were weighed and normalized to tibia length (HW/TL) as a measure of hypertrophy and were then preserved in 10% formalin solution for histology studies. Sham (*n* = 8) and VNS (*n* = 6) hearts were embedded in paraffin, cut into 5 μm thick cross-sectional slices, and stained with Masson’s Trichrome. The stained heart slices were imaged using Nikon microscope (Model ECLIPSE Ni-E, Nikon Corporation, Tokyo, Japan) system at 4× magnification. NIS-Elements AR (version 4.51.01) software was used to scan each slide and export the images for post-processing.

Cardiac measures, including left ventricle and right ventricle free wall thickness, septum thickness and cross-section diameter, were measured for both Sham and VNS rats. All thickness measures performed on the cardiac cross sections were comprised of an average of ten measurements. Finally, cardiac fibrosis was quantified using ImageJ software (developed by Wayne Rasband, National Institutes of Health, United States) to isolate the blue fibrotic regions of the cardiac slices. As the time point of the heart extraction varied between rats, the gross morphology and histology measures were plotted separately for Sham and VNS rats with respect to survival times and fit with a linear regression.

### Statistics

BP and HR data are presented as mean ± SE. Cumulative event-free survival is presented using Kaplan-Meier curves, and the Log-Rank test was used to determine statistical significance. The acute effects of VNS, measured as absolute values at different time intervals, were compared using repeated measures one-way ANOVA with Tukey’s multiple comparisons correction, with an adjusted value *P* < 0.05 indicating statistical significance. The linear regression slopes for the longitudinal BP and HR measures were compared between Sham and VNS rats using one-way ANOVA. The circadian rhythm parameters were compared between Sham and VNS rats and Week 6 and Week 9 time points using two-way ANOVA with Tukey’s multiple comparisons correction. Finally, the structural effects were compared using simple linear regressions to compare structural measures as a function of survival for Sham and VNS rats. Statistical significance was determined at *P* < 0.05.

## Results

### Baseline Parameters

Baseline ECG (HR and HRV) and BP (MAP, SBP, DBP, and PP) parameters show no significant differences between Sham and VNS rats (see Supplementary Table S2). Specifically, Baseline HR (Sham: 386 ± 5 bpm and VNS: 389 ± 5 bpm) and SBP (Sham: 192 ± 10 mmHg and VNS: 187 ± 8 mmHg) were similar between groups and are consistent with previously published parameters in hypertensive rats (Annoni et al., 2015).

### Event-Free Survival Assessment

Figure 1B shows the cumulative event-free survival rates of the Sham and VNS rats, with a significant improvement in survival of VNS rats (*P* < 0.05). The median survival in VNS rats increased from 9.1 (95% CI: 7.7 to 10.7) weeks for Sham rats to 12 (95% CI: 10.3 to 13.6) weeks for VNS rats. In addition, 2/9 VNS rats survived to the end of the study, whereas 0/8 Sham rats survived. The relationship between various Baseline ECG (HR and HRV) and BP (MAP, SBP, DBP, and PP) parameters and survival time are shown in Supplementary Figure S1, demonstrating that none of Baseline parameters significantly influenced the difference in survival rates observed in Sham and VNS rats.

To better understand possible factors contributing to the prolonged survival of VNS rats, we further evaluated the effects of VNS therapy on (1) acute cardiovascular and hemodynamic response, (2) chronic disease progression, (3) autonomic tone, and (4) structural properties of the heart.

### Impact of VNS Cycles on Acute Cardiovascular and Hemodynamic Response

At Week 9, we quantified the effect of acute VNS on the cardiovascular and hemodynamic response by evaluating various BP and ECG parameters during the “Pre,” “VNS On,” “Post 1,” and “Post 2” intervals. Figure 2 shows the percent change in SBP, BPV, contractility, HR and HRV with respect to the “Pre” interval values. Both SBP and contractility slightly decrease during the “VNS On” interval, which is significantly lower with respect to the “Post 2” interval, where a slight overshoot from pre-stimulation values was observed. In contrast, HRV and BPV were significantly increased during “VNS On” with respect to both pre- and post-stimulation values, and immediately returned to pre-stimulation values after the cessation of therapy. However, HR was not significantly affected by the low-level VNS therapy.

Similar acute analysis was performed for the VNS rats at Week 12 and is shown in Supplementary Figure S2. No significant differences were observed in SBP, BPV, HR, or contractility values. However, HRV was significantly increased during the “VNS On” interval in comparison to the “Post 2” interval.

### Impact of Chronic, Cyclic VNS on HTN Progression

**FIGURE 3:**
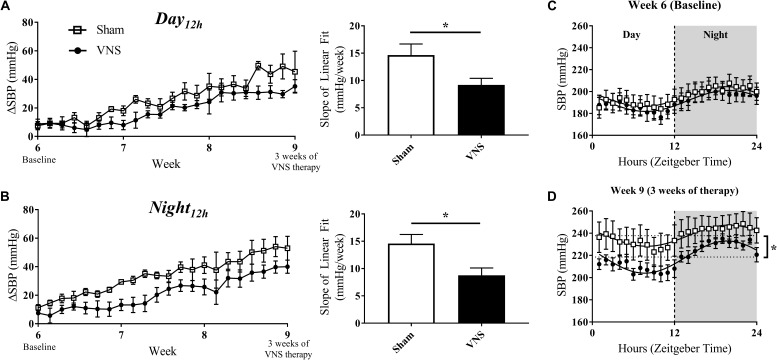
Relative changes in SBP (ΔSBP) as HTN progresses. ΔSBP as a function of time and slopes of the linear regressions are shown for Sham and VNS rats during the **(A)** *Day*_12h_ and **(B)** *Night*_12h_ intervals. Circadian rhythm of SBP for Sham and VNS rats are shown for Week 6 **(C)** and Week 9 **(D)**. ^∗^Indicates statistical significance between Sham and VNS rats.

HTN progression in the Sham and VNS rats was quantified by the relative change in SBP (ΔSBP) and HR (ΔHR) over time with respect to Baseline values during the *Day*_12h_ and *Night*_12h_ intervals. Figure 3A,B (left panels) shows that the progressive increase of ΔSBP due to HTN is attenuated in the VNS rats compared to the Sham rats, as is suggested by the growing separation in the ΔSBP curves over time. Indeed, the linear regression slopes in the Sham rats are significantly larger when compared to VNS rats during both the *Day*_12h_ (Sham: 14.6 ± 2.0 mmHg/week; VNS: 9.2 ± 2.3 mmHg/week; *P* < 0.05) and *Night*_12h_ (Sham: 14.6 ± 1.7 mmHg/week; VNS: 8.8 ± 1.4 mmHg/week; *P* < 0.05) intervals.

The circadian rhythm was evaluated at Week 6 and Week 9 (Figure 3C,D, respectively). While similar circadian rhythm characteristics were observed for both Sham and VNS rats at Week 6, three weeks of VNS therapy resulted in a significantly decreased average baseline (*MESOR*) value in VNS rats in comparison to the Sham rats (see ^∗^ in Figure 3D), providing further evidence of an attenuation in the rise in SBP due to VNS.

The effects of disease progression in the Sham and VNS rats on other hemodynamic parameters (MAP, DBP, and PP) are shown in Supplementary Figures S3–S5. VNS rats experienced a significant attenuation in ΔMAP during the *Night*_12h_ but not during the *Day*_12h_ interval (Supplementary Figures S3A,B). However, ΔDBP and ΔPP values did not show any significant changes during either interval (Supplementary Figures S4, S5A,B). In addition, circadian rhythm analysis for MAP, DBP, and PP demonstrated similar cosinor characteristics in Sham and VNS rats at Week 6. In contrast, at Week 9, the *MESOR* values were significantly lower in VNS rats, providing further evidence of the attenuation in progression of HTN due to VNS therapy.

**FIGURE 4:**
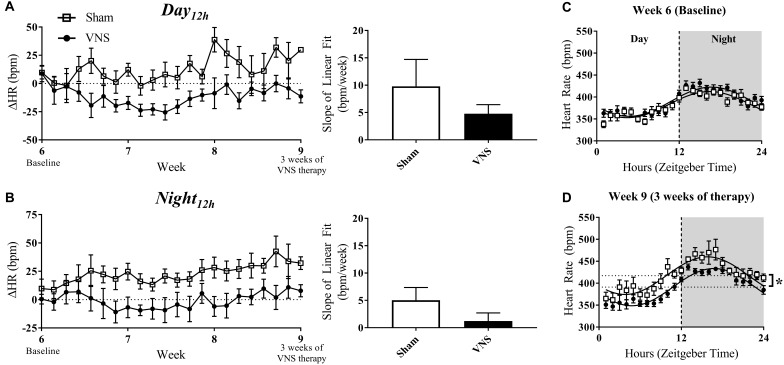
Relative changes in HR (ΔHR) as HTN progresses. ΔHR as a function of time and slopes of the linear regressions are shown for Sham and VNS rats during the **(A)** *Day*_12h_ interval and **(B)** *Night*_12h_ intervals. Circadian rhythm analysis of HR for Sham and VNS rats are shown for Week 6 **(C)** and Week 9 **(D)**. ^∗^Indicates statistical significance between Sham and VNS rats.

Figure 4A,B (left panel) quantifies the relative change in HR over time during the *Day*_12h_ and *Night*_12h_ intervals in the Sham and VNS rats. The slopes of the ΔHR linear regression, although lower in VNS rats, were not significantly different during either interval (Figure 4A,B, right panel). Circadian rhythm of HR showed no difference between Sham and VNS rats at Week 6 (Figure 4C), but after 3 weeks of VNS therapy, the *MESOR* value for Sham rats was significantly higher compared to VNS rats (see ^∗^ in Figure 4D).

### Impact of VNS on Autonomic Regulatory Function

**FIGURE 5:**
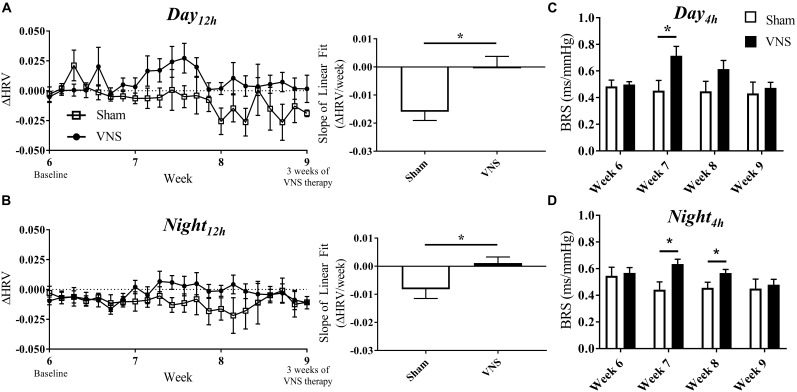
Relative changes in HRV (ΔHRV) as HTN progresses. ΔHRV as a function of time and slopes of the linear regressions are shown for Sham and VNS rats during the **(A)** *Day*_12h_ interval and **(B)** *Night*_12h_ intervals. BRS was quantified during the *Day*_4h_ **(C)** and *Night*_4h_ **(D)** intervals for Week 6 through Week 9. ^∗^Indicates statistical significance between Sham and VNS rats.

The effects of VNS on autonomic tone were evaluated through HRV and BRS measures and are presented in Figure 5. The relative change in HRV during *Day*_12h_ and *Night*_12h_ intervals are shown for Sham and VNS rats in Figure 5A,B, respectively. The results show that VNS significantly affects the dynamics of HRV over time. Specifically, ΔHRV was maintained or slightly increased in VNS rats as opposed to the Sham rats, which experienced negative ΔHRV as HTN progressed. Indeed, the slopes of the ΔHRV linear regression were significantly decreased over 3 weeks of therapy for Sham (*Day*_12h_: -1.5 ± 0.3%; *Night*_12h_: -0.8 ± 0.3%) but not for VNS rats, where ΔHRV remained relatively stable over time (*Day*_12h_: 0.01 ± 0.3%; *Night*_12h_: 0.1 ± 0.2%). Finally, Figure 5C,D shows BRS values measured weekly from Week 6 to Week 9, showing significantly increased BRS values in VNS rats compared with Sham rats at Week 7 during both *Day*_4h_ and *Night*_4h_ and at Week 8 during the *Night*_4h_ interval. This acute increase in BRS for VNS rats was not present at Week 9, where BRS values were similar between VNS and Sham rats.

### Impact of VNS on Structural Changes in the Hypertensive Heart

**FIGURE 6:**
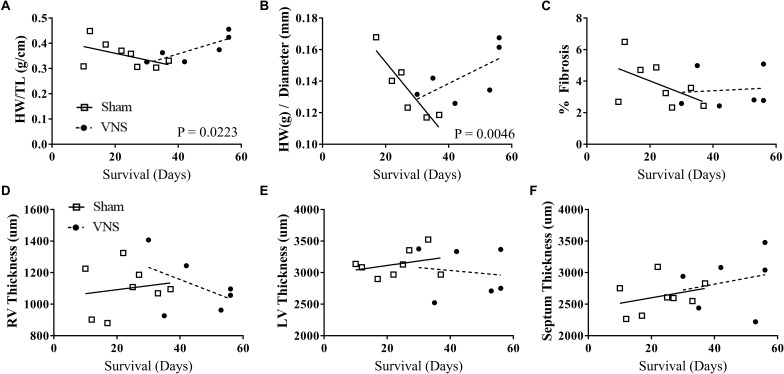
Gross morphology and histology studies for Sham (*n* = 8) and VNS (*n* = 6) hypertensive rats. The effects of VNS on cardiac structural properties – **(A)** HW/TL, **(B)** HW/diameter, **(C)** percent fibrosis, **(D)** right ventricle (RV) and **(E)** left ventricle (LV) free wall thickness, and **(F)** septum thickness – as a function of survival rate along with corresponding linear regression fits.

The results of the gross morphology and histology studies are shown in Figure 6 as a function of survival rate along with the corresponding linear regression values from Sham and VNS rats. Examples of stained cardiac cross-sections for Sham and VNS rats are shown in Supplementary Figure S6. Figure 6A shows the relationship of HW/TL over time, which was significantly different between groups. Sham rats had a decrease in HW/TL (-0.0027 ± 0.0019 g/cm/day) while VNS rats exhibited an increase in HW/TL as a function of survival time (0.0038 ± 0.0013 g/cm/day; *P* < 0.05). In addition, HW/diameter (Figure 6B) showed a similar result with Sham rats having a decrease in HW/diameter over time (-0.0024 ± 0.0006 g/cm/day), while VNS rats showing an increase over time (0.0001 ± 0.0006 g/cm/day; *P* < 0.05). The raw data for the structural changes in Sham and VNS hypertensive hearts are shown in Supplementary Table S5. Although changes were observed in HW, individual thickness measures of the RV and LV free wall as well as septum showed no significant differences between treatment groups (Figure 6D–F). In addition, no changes were seen in diameter or body weight between Sham and VNS rats as a function of survival (Supplementary Figure S6). Finally, chronic VNS therapy resulted in no changes in the percent of fibrosis in the cardiac cross-sections as seen in Figure 6C.

## Discussion

The major finding of this study was a significant improvement in the long-term survival of hypertensive rats treated with VNS therapy, with median event-free survival increasing by 78% for VNS rats in comparison to Sham rats. This study further evaluated the effects of VNS on various cardiovascular, hemodynamic, and structural cardiac properties that may contribute to prolonged survival of VNS rats with respect to Sham rats. Our results can be summarized as follows: (1) acute VNS improved autonomic balance by significantly increasing HRV during stimulation, which may lead to beneficial effects observed in the chronic use of VNS therapy, (2) chronic VNS slowed the progression of HTN through and an attenuation in the rise of SBP, demonstrated in the linear regression analysis during the day and night intervals and the baseline shift in circadian rhythm, (3) chronic VNS preserved autonomic balance as HTN progressed by maintaining elevated HRV and BRS measures in the VNS rats compared to Sham rats, and (4) VNS significantly altered cardiac structure increasing heart weight, but did not alter the amount of fibrosis in the hypertensive hearts.

### Acute Hemodynamic Response

Low-level VNS, here, is defined by the magnitude stimulation and subsequent impact on heart rate. Previous research has looked at various current amplitudes to classify VNS intensity with high intensity causing a greater than 60% reduction in HR and medium intensity stimulation causing a 40% or less reduction in HR (Zhang et al., 2009). Here the initial titration for the VNS rats was established to elicit no more than a 10% drop in HR during the day time. As a result, there were no significant changes in mean HR while VNS was on. However, this effect can be controlled by altering stimulation parameters as shown previously (Ojeda et al., 2016; Stauss, 2017).

The acute analysis at Week 9 revealed a significant decrease in BP and contractility in response to VNS that returned to pre-stimulation levels at the cessation of therapy. This effect is likely mediated through activation of the aortic depressor nerve which sends signals to the brain resulting in the activation of the baroreflex and a decrease in BP (Plachta et al., 2014). This acute BP reduction has been demonstrated previously where selective and non-selective VNS therapy was applied in anesthetized rats (Plachta et al., 2014, 2016).

VNS continued to show a strong acute effect on HRV even after 3 weeks of therapy, increasing HRV as stimulation was applied. In addition to changes in HRV, BPV was also significantly increased, but to a lesser extent. This result has been observed previously as BPV reflexively increases acutely in response to vagal activation (Clement et al., 1984; Annoni and Tolkacheva, 2018). The acute change in HRV is evident of enhanced parasympathetic activity or decreased sympathetic activity, which may provide an additive effect over time and improve autonomic balance. This acute change in HRV was also observed at Week 12, after 6 weeks of therapy, indicating that low-level intermittent VNS was still influencing HR dynamics.

### Chronic Disease Progression

VNS therapy has been demonstrated to have beneficial long-term effects in HTN animal models. Our previous chronic study demonstrated the impact of low-level intermittent VNS on BP through a long-term effect resulting in a reduction in the spontaneous rise in BP after 4 weeks of therapy in hypertensive rats (Annoni et al., 2015). Here, a significant attenuation was observed after just 3 weeks of VNS therapy. This attenuation in rising SBP by Week 9 may play a role in the delay in the onset of adverse events in hypertensive rats.

The characteristics of BP control in Sham and VNS rats were further evaluated using cosinor analysis to compare circadian rhythms in SBP and HR, which are mediated by cyclic variations in autonomic activity (White, 2007). Although there was a significant increase in the *MESOR* value for Sham in both HR and SBP, the amplitude and acrophase of the cosinor parameters were not significantly different. In addition, this abnormality in circadian rhythm is associated with an increased risk of cardiovascular risk and target organ injury (White, 2000). Through stimulation of the parasympathetic nervous system, VNS may help maintain regular cyclic BP control.

### Autonomic Regulatory Function

Heart rate variability is commonly used in clinical practice as a prognostic indicator of cardiovascular conditions. In the case of HTN and heart failure, autonomic dysfunction results in decreased HRV. It is postulated that stimulation of the parasympathetic nervous system, through right cervical VNS, has the potential to rebalance the autonomic nervous system by directly increasing parasympathetic activity and reflexively reducing sympathetic activity. Indirect measures of autonomic balance, such as HRV and BRS, have been assessed in numerous studies showing a restoration of autonomic balance and improved prognostic indications for the VNS-treated group (Zhang et al., 2009; Saku et al., 2014). Here, we demonstrate results consistent with those previously seen. VNS preserved HRV values from Week 6 through Week 9, while Sham rats experienced a large reduction in HRV during both the day and night intervals. Our results also showed an acute increase in BRS during Weeks 7 and 8, indicating improved BP and HR control during this time. In HTN, chronic structural changes in the heart and vasculature alter the function of the arterial baroreceptors decreasing their sensitivity to fluctuations in BP, ultimately decreasing BRS (Chapleau, 2012). Overall, the beneficial effects on HRV and BRS in the VNS rats may be mediated through balancing autonomic activation and altering structural changes in vasculature, which may ultimately contribute to the prolonged survival in VNS rats.

### Structural Cardiac Effects

Our results demonstrate that VNS had a significant impact on hypertrophy with increased heart weight as survival times increased in hypertensive rats. In addition, opposite trends in LV and RV free wall thicknesses were observed, with VNS showing a reduction over time. However, there is no change in the amount of fibrosis between Sham and VNS rats. Although, without sufficient overlap of Sham and VNS data at early and late time points in the survival curve, assessing the progression of structural changes in the treated and untreated heart is challenging. In addition, the increased survival of VNS rats means these rats were older and exposed to high-salt diet for a longer duration at the time the hearts were analyzed, which may influence the results observed in this study.

VNS may be altering the progression of cardiac remodeling in response to increasing BP levels. Previously, Dahl salt-sensitive rats have been shown to advance to later stages of HTN rapidly while on 8% NaCl with significant hypertrophy and LV dilation as early as 11 and 15 weeks of age, respectively (Inoko et al., 1994). With respect to our experimental design, those time points correspond to Week 6 and Week 10. VNS therapy may delay or alter the time course of hypertensive heart disease in these rats. Another important factor to consider is the advanced-stage of HTN in the rats in this study. Nguyen et al. (2016) demonstrated that even in the early stages of HTN, the heart undergoes structural and electrical remodeling. The rats in this study had well established HTN at the beginning of VNS therapy and were continued on a high salt diet for the remainder of the study. Intervening in the earlier stages of HTN may provide restorative or preventative structural cardiac effects. Further studies are necessary to fully understand the effects of VNS therapy on hypertensive heart disease in various stages of HTN.

Numerous other mechanisms of VNS therapy have been evaluated in HTN and cardiovascular disease models which could be contributing to the improved survival, attenuation in the spontaneous rise in BP, and improved autonomic tone observed in this study. Although the change in BP observed in this study is small, the survival was significantly improved in the VNS rats, which suggests other mechanisms may be contributing. Additional mechanisms through which VNS has been hypothesized to provide therapeutic effects include anti-adrenergic effects, anti-inflammatory pathways, activation of nitric oxide release in the heart, and inhibition of the renin-angiotensin system. Suppressing central sympathetic outflow through activation of vagal nerve afferent fibers can lead to a reduction in sympathetic nervous system activity, resulting in reductions in HR, vascular resistance, and arterial BP. Several studies have demonstrated the ability of VNS to reduce systemic inflammation through the activation of the cholinergic anti-inflammatory pathway, reducing cytokine synthesis and the inflammatory response (Tracey, 2007; Zhang et al., 2009). Inflammation is associated with HTN, although the cause-and-effect relationship still remains unclear. It is hypothesized that inflammation can lead to endothelial dysfunction and increased oxidative stress, which promote the development and progression of HTN (Dinh et al., 2014). VNS has also been shown to directly activate the nitric oxide pathway producing nitric oxide in the ventricles, altering cardiac electrophysiology and decreasing arrhythmia susceptibility (Brack et al., 2009). Another proposed mechanism of VNS is inhibition of the renin-angiotensin system through afferent vagal nerves, resulting in a reduction in renin in the kidneys and marked reduction in plasma angiotensin II levels (Mancia et al., 1975; Zhang et al., 2009).

The model used in the study progressed quickly toward HTN and resulted in a large drop off with the Sham and VNS rats. Previously, a similar model was used to evaluate the effect of 4 weeks of VNS therapy in HTN rats (Annoni et al., 2015). Surprisingly, all rats survived in that study, which might be partially explained by slight difference in the stimulation parameters, and partly attributed to the model variability. Despite the severe stage of HTN, there is still a significant impact of VNS on the progression of HTN in these rats. Further studies in hypertensive rats could use a lower dose of salt, resulting in a slower progression of HTN and a lower dropout rate. In the slower developing models, VNS may have a similar, if not stronger, impact on the disease and overall survival. Another important factor of this study was the stimulation parameters such as pulse width, frequency, amplitude, and duty cycle, which can significantly influence the acute and chronic effects of VNS. However, stimulation parameters have not been optimized for treating HTN and could be further evaluated to determine which parameters result in beneficial changes in BP and HR.

### Limitations

This study has several limitations. First, there were nine and eight rats in the VNS and Sham groups, respectively, and they were in advanced stages of HTN by Week 6 when therapy was initiated for VNS rats. In our chronic analysis, one of the limitations is that as the study advances, the rats with the highest BP reach end points earlier, which may lead to underestimated group averages of SBP. On average, the Sham rats reached an endpoint prior to the VNS rats. For the acute analysis, one of the limitations is the lack of *direct* measurements of the autonomic tone. Indeed, the design of our study only allow for *indirect* assessment of the autonomic tone via various measurements (HRV, BRS, and BPV). Moreover, we had to rely on published literature to provide interpretation of such indirect parameters. Another limitation is the amount of analyses performed within the scope of this study. Continuous data was collected from the HTN rats which will require additional analyses of the BP and ECG waveforms to further evaluate the effects of VNS therapy. Acute effects and autonomic evaluation through BRS measures was limited to 4-h data segments during day and night intervals. Future work will include quantifying these parameters over larger data segments to better understand the progression due to HTN as well as changes due to variation in autonomic tone based on time of day. Further studies need to be conducted to evaluate the effects of VNS therapy in various stages of HTN to investigate the preventative and restorative potential of VNS in controlling BP and addressing adverse cardiovascular effects associated with HTN.

## Conclusion

Overall, our results are consistent with previous studies that show potential for VNS to provide therapy for HTN and HTN-induced heart disease. This study shows a strong beneficial effect of VNS therapy on long-term survival, BP control, and autonomic tone. These results motivate additional studies to investigate the mechanism of VNS in treating HTN and determine optimal stimulation parameters for therapeutic effects.

## Author Contributions

EA designed and performed experiments, completed the data collection and analysis, and wrote the manuscript. DVH performed experiments including surgeries and animal care. YG and BL performed data collection and analysis. IL, BK, and JO supervised the project and reviewed the manuscript. ET designed experiments, supervised data analysis, and assisted in preparation of the manuscript. All authors reviewed the manuscript.

## Conflict of Interest Statement

IL and BK are employees of LivaNova. The remaining authors declare that the research was conducted in the absence of any commercial or financial relationships that could be construed as a potential conflict of interest.

## References

[B1] Annoni E. M. Tolkacheva E. G. (2018). “Acute hemodynamic effects of vagus nerve stimulation in conscious hypertensive rats,” in *Paper Presented at the 40th Annual International Conference of the IEEE-Engineering-in-Medicine-and-Biology-Society (EMBC)*, Honolulu, HI. 10.1109/EMBC.2018.8513025 30441172

[B2] Annoni E. M. Xie X. Lee S. W. Libbus I. KenKnight B. H. Osborn J. W. (2015). Intermittent electrical stimulation of the right cervical vagus nerve in salt-sensitive hypertensive rats: effects on blood pressure, arrhythmias, and ventricular electrophysiology. *Physiol. Rep.* 3:e12476. 10.14814/phy2.12476 26265746 PMC4562562

[B3] Bertinieri G. Di Rienzo M. Cavallazzi A. Ferrari A. Pedotti A. Mancia G. (1985). A new approach to analysis of the arterial baroreflex. *J. Hypertens. Suppl.* 3 S79–S81. 2856787

[B4] Brack K. E. Patel V. H. Mantravardi R. Coote J. H. Ng G. A. (2009). Direct evidence of nitric oxide release from neuronal nitric oxide synthase activation in the left ventricle as a result of cervical vagus nerve stimulation. *J. Physiol.* 587 3045–3054. 10.1113/jphysiol.2009.169417 19403619 PMC2718260

[B5] Chapleau M. W. (2012). “Baroreceptor reflexes,” in *Primer on the Autonomic Nervous System*, 3rd Edn, eds Robertson D. Biaggioni I. Burnstock G. Low P. A. Paton J. F. R. (Amsterdam: Academic Press).

[B6] Chapleau M. W. Rotella D. L. Reho J. J. Rahmouni K. Stauss H. M. (2016). Chronic vagal nerve stimulation prevents high-salt diet-induced endothelial dysfunction and aortic stiffening in stroke-prone spontaneously hypertensive rats. *Am. J. Physiol. Heart Circ. Physiol.* 311 H276–H285. 10.1152/ajpheart.00043.2016 27208157 PMC4967207

[B7] Clement D. Jordaens L. Heyndrickx G. (1984). Influence of vagal nervous activity on blood pressure variability. *J. Hypertens. Suppl.* 2 S391–S393. 6599687

[B8] Dinh Q. N. Drummond G. R. Sobey C. G. Chrissobolis S. (2014). Roles of inflammation, oxidative stress, and vascular dysfunction in hypertension. *Biomed Res. Int.* 2014:406960. 10.1155/2014/406960 25136585 PMC4124649

[B9] Egan B. M. Zhao Y. Axon R. N. Brzezinski W. A. Ferdinand K. C. (2011). Uncontrolled and apparent treatment resistant hypertension in the United States, 1988 to 2008. *Circulation* 124 1046–1058. 10.1161/CIRCULATIONAHA.111.030189 21824920 PMC3210066

[B10] Fields L. E. Burt V. L. Cutler J. A. Hughes J. Roccella E. J. Sorlie P. (2004). The burden of adult hypertension in the United States 1999 to 2000 - A rising tide. *Hypertension* 44 398–404. 10.1161/01.hyp.0000142248.54761.56 15326093

[B11] Guyenet P. G. (2006). The sympathetic control of blood pressure. *Nat. Rev. Neurosci.* 7 335–346. 10.1038/nrn1902 16760914

[B12] Huang J. Qian J. Yao W. Wang N. Zhang Z. Cao C. (2015). Vagus nerve stimulation reverses ventricular electrophysiological changes induced by hypersympathetic nerve activity. *Exp. Physiol.* 100 239–248. 10.1113/expphysiol.2014.082842 25720663

[B13] Inoko M. Kihara Y. Morii I. Fujiwara H. Sasayama S. (1994). Transition from compensatory hypertrophy to dilated, failing left ventricles in Dahl salt-sensitive rats. *Am. J. Physiol. Heart Circ. Physiol.* 267 H2471–H2482. 10.1152/ajpheart.1994.267.6.H2471 7810745

[B14] Lalande S. Johnson B. D. (2008). Diastolic dysfunction: a link between hypertension and heart failure. *Drugs Today* 44 503–513. 10.1358/dot.2008.44.7.1221662 18806901 PMC2713868

[B15] Lee S. W. Li Q. Libbus I. Xie X. KenKnight B. H. Garry M. G. (2016). Chronic cyclic vagus nerve stimulation has beneficial electrophysiological effects on healthy hearts in the absence of autonomic imbalance. *Physiol. Rep.* 4:e12786. 10.14814/phy2.12786 27173672 PMC4873636

[B16] Li P. Nader M. Arunagiri K. Papademetriou V. (2016). Device-based therapy for drug-resistant hypertension: an update. *Curr. Hypertens. Rep.* 18 1–12. 10.1007/s11906-016-0671-4 27402013

[B17] Mancia G. De Backer G. Dominiczak A. Cifkova R. Fagard R. Germano G. (2007). 2007 Guidelines for the management of arterial hypertension - The task force for the management of arterial hypertension of the European society of hypertension (ESH) and of the European society of cardiology (ESC). *Eur. Heart J.* 28 1462–1536. 10.1093/eurheartj/ehm236 17562668

[B18] Mancia G. Grassi G. (2014). The autonomic nervous system and hypertension. *Circ. Res.* 114 1804–1814. 10.1161/CIRCRESAHA.114.302524 24855203

[B19] Mancia G. Romero J. C. Shepherd J. T. (1975). Continuous inhibition of renin release in dogs by vagally innervated receptors in the cardiopulmonary region. *Circ. Res.* 36 529–535. 10.1161/01.RES.36.4.529 1116245

[B20] NCD Risk Factor Collaboration [NCD-RisC] (2017). Worldwide trends in blood pressure from 1975 to 2015: a pooled analysis of 1479 population-based measurement studies with 19 1 million participants. *Lancet* 389 37–55. 27863813 10.1016/S0140-6736(16)31919-5 PMC5220163

[B21] Nguyen T. P. Sovari A. A. Pezhouman A. Iyer S. Cao H. Ko C. Y. (2016). Increased susceptibility of spontaneously hypertensive rats to ventricular tachyarrhythmias in early hypertension. *J. Physiol.* 594 1689–1707. 10.1113/JP271318 26775607 PMC4799964

[B22] Ojeda D. Le Rolle V. Romero-Ugalde H. M. Gallet C. Bonnet J.-L. Henry C. (2016). Sensitivity analysis of vagus nerve stimulation parameters on acute cardiac autonomic responses: chronotropic, inotropic and dromotropic effects. *PLoS One* 11:e0163734. 10.1371/journal.pone.0163734 27690312 PMC5045213

[B23] Plachta D. T. Gierthmuehlen M. Cota O. Espinosa N. Boeser F. Herrera T. C. (2014). Blood pressure control with selective vagal nerve stimulation and minimal side effects. *J. Neural Eng.* 11:036011. 10.1088/1741-2560/11/3/036011 24809832

[B24] Plachta D. T. Zentner J. Aguirre D. Cota O. Stieglitz T. Gierthmuehlen M. (2016). Effect of cardiac-cycle-synchronized selective vagal stimulation on heart rate and blood pressure in rats. *Adv. Ther.* 33 1246–1261. 10.1007/s12325-016-0348-z 27220533

[B25] Saku K. Kishi T. Sakamoto K. Hosokawa K. Sakamoto T. Murayama Y. (2014). Afferent vagal nerve stimulation resets baroreflex neural arc and inhibits sympathetic nerve activity. *Physiol. Rep.* 2:e12136. 10.14814/phy2.12136 25194023 PMC4270242

[B26] Stauss H. M. (2017). Differential hemodynamic and respiratory responses to right and left cervical vagal nerve stimulation in rats. *Physiol. Rep.* 5:e13244. 10.14814/phy2.13244 28400500 PMC5392529

[B27] Tracey K. J. (2007). Physiology and immunology of the cholinergic antiinflammatory pathway. *J. Clin. Investig.* 117 289–296. 10.1172/jci30555 17273548 PMC1783813

[B28] White W. B. (2000). Ambulatory blood pressure monitoring: dippers compared with non-dippers. *Blood Press. Monit.* 5 S17–S23. 10.1097/00126097-200005001-00004 10904238

[B29] White W. B. (2007). Importance of blood pressure control over a 24-hour period. *J. Manag. Care Pharm.* 13(8 Suppl. B), 34–39. 10.18553/jmcp.2007.13.s8-b.34 17970615 PMC10437563

[B30] World Health Organization [WHO] (2013). *Global Brief on Hypertension.* Geneva: World Health Organization.

[B31] Xie X. Lee S. W. Johnson C. Ippolito J. KenKnight B. H. Tolkacheva E. G. (2014). “Intermittent vagal nerve stimulation alters the electrophysiological properties of atrium in the myocardial infarction rat model,” in *Paper Presented at the 36th Annual International Conference of the IEEE-Engineering-in-Medicine-and-Biology-Society (EMBC)*, Chicago, IL. 10.1109/EMBC.2014.6943904 25570272

[B32] Zhang Y. Popovic Z. B. Bibevski S. Fakhry I. Sica D. A. Van Wagoner D. R. (2009). Chronic vagus nerve stimulation improves autonomic control and attenuates systemic inflammation and heart failure progression in a canine high-rate pacing model. *Circ. Heart Fail.* 2 692–699. 10.1161/circheartfailure.109.873968 19919995

