# Supplementary material for: Chronic Low-Level Vagus Nerve Stimulation Improves Long-Term Survival in Salt-Sensitive Hypertensive Rats
Source: Front Physiol. 2019 Jan 31;10:25. doi: 10.3389/fphys.2019.00025 (PMC6365472; doi:10.3389/fphys.2019.00025)
Supplement: Supplementary file 1 [file Data_Sheet_1.docx]

Supplementary Material

**Chronic low-level vagus nerve stimulation improves long-term survival in salt-sensitive hypertensive rats**

Elizabeth M. Annoni^1^, Dusty Van Helden^2^, Yugene Guo^3^, Brett Levac^4^, Imad Libbus^5^, Bruce H. KenKnight^5^, John W. Osborn^2^, Elena G. Tolkacheva*^1^

*** Correspondence:** Elena G. Tolkacheva: talkacal@umn.edu

# Methods

## Surgical Procedures

Prophylactic antibiotic (gentamicin sulphate; 10 mg/kg, i.m.) was given prior to each surgery. During the surgeries, rats were anaesthetized with isoflurane (5% for induction, 2% or 3.5% for maintenance) in oxygen (2 L/min for induction, 1 L/min for maintenance). The rat’s body temperature was maintained at 37ºC on a temperature-controlled surgery table.

### Vagus Nerve Stimulator Implantation

The VNS pulse generator and cuff electrodes were implanted as described previously.(Shinlapawittayatorn et al., 2014; Xie et al., 2014) Briefly, the surgical regions on the back and the neck were shaved. Bipolar cuff electrodes were implanted through a small incision on the neck and placed around the right cervical vagus nerve and common carotid artery bundle. The pulse generator was implanted subcutaneously and positioned on the back of the rat. VNS was applied with a pulse frequency of 20 Hz, a pulse width of 500 μs, and an output current of 0.25 - 1.0 mA. The amplitude of the current was selected based on a stimulation level of sufficient strength to appear on the ECG but without causing an acute peak reduction in HR greater than 10% during the active phase of the stimulation cycle. Each VNS cycle consisted of an active phase, consisting of 14 seconds of full strength stimulation plus a 2-second ramp up and ramp down phase, followed by an inactive phase, 62 seconds in duration, resulting in a duty cycle of 22.5%. The intermittent VNS stimulation was applied in VNS rats throughout the entire study, starting at Week 6, both at day and night. VNS stimulation amplitude was checked weekly to ensure the VNS stimulation was still appearing on the ECG trace and lowering HR, but maintained less than 10% decrease in HR, however no changes in amplitude were needed. All other parameters were held constant throughout the study.

### *DSI Telemeter Implantation*

DSI transmitters were implanted as described previously.(Veitenheimer & Osborn, 2011) Briefly, the chest and inner thigh were shaved and the transmitter pressure catheter was implanted through a small incision on the inner left hind limb into the descending aorta via the left femoral artery. The two ECG leads were fixed subdermally on the chest muscle. After surgery, the rats were individually housed in cages where they were able to freely move about while receivers (model RPC1, DSI Inc.) connected to a computer via a Data Exchange Matrix (MX2, DSI Inc.) continuously collected data from each rat. The ECG and arterial BP data were collected at a sampling rate of 500 Hz throughout the study.

## Data Analysis

**Supplementary Table 1.** Summary of data analyses performed including the time points and number of rats included in the analysis.

|  | **Time Point** | | | | |
| --- | --- | --- | --- | --- | --- |
| **Analysis** | Week 6 | Week 7 | Week 8 | Week 9 | Week 12 |
| Acute VNS Response  4-h segment (***Day_4h_*** and ***Night_4h_***)  VNS Rats Only* | VNS (n = 6)^†^ |  |  | VNS (n = 6)  Figure S2 | VNS (n = 2)  Figure S3 |
| Longitudinal Analysis  SBP, MAP, DBP, PP, HR, HRV  12-h segment (***Day_12h_*** and Night***_12h_***) | Analysis uses all data from all rats and time points  Figures 3-5;  Figure S3-S5 | | | | |
| Circadian Rhythm  24-h segment | VNS (n = 9)  Sham (n = 8)  Figures 3-5; S3-S5 |  |  | VNS (n = 8)  Sham (n = 5)  Figures 3-5; S3-S5 |  |
| BRS  4-h segment (***Day_4h_*** and ***Night_4h_***) | VNS (n = 9)  Sham (n = 8)  Figure 5 | VNS (n = 9)  Sham (n = 8)  Figure 5 | VNS (n = 9)  Sham (n = 6)  Figure 5 | VNS (n = 8)  Sham (n = 5)  Figure 5 |  |

* Only VNS rats with adequate ECG signal to identify stimulation intervals were used for analysis

† Data published in [23]

### Acute effects of VNS therapy

In the acute window analysis, “Pre” was defined as the 7-second segment prior to the VNS therapy. “VNS On” was defined as the 14 seconds during which VNS was applied, with the exclusion of the 2-second ramp up and ramp down intervals. “Post 1” and “Post 2” were defined as two consecutive 7-second segments immediately after VNS stimulation. Due to the noisy nature of the 2-lead ECG data, HR was derived using pulse interval calculated from the derivative of the BP trace, as described in ^23^.

### Baroreflex Sensitivity

The time domain baroreflex sensitivity (BRS) sequence method quantifies concordant changes in BP and HR. BRS analysis was analyzed from Week 6 to Week 9. The parameters for the BRS method were set to the following: a minimum sequence length of 16 consecutive beats, a minimum change in BP of 5 mmHg, and a R^2^ value threshold of 0.85.

# Supplementary Results

## Baseline parameters

**Supplementary Table 2.** Baseline parameters, presented as mean +/- standard error, for VNS (n = 9) and Sham (n = 8) rats calculated as a three-day average prior to the start of VNS therapy.

| **Baseline Parameter** | **Sham (n = 8)** | **VNS (n = 9)** | **P-value** |
| --- | --- | --- | --- |
| HR (bpm) | 386 ± 5 | 389 ± 5 | 0.52 |
| HRV (%) | 9.5 ± 0.6 | 9.8 ± 0.3 | 0.55 |
| SBP (mmHg) | 192 ± 10 | 187 ± 8 | 0.65 |
| MAP (mmHg) | 160 ± 9 | 155 ± 7 | 0.66 |
| DBP (mmHg) | 131 ± 9 | 128 ± 6 | 0.7 |
| PP (mmHg) | 61 ± 2 | 59 ± 3 | 0.63 |

Individual baseline parameters for Sham and VNS rats were plotted versus survival time, in days, to observe whether any baseline parameters factored into the survival times observed in the two groups (Figure S1). All parameters showed similar ranges in both Sham and VNS rats at baseline, and did not contribute significantly to the difference observed in survival. This was evaluated using Cox Proportional Hazards test where baseline parameters along with treatment group were evaluated as factors. The results of the test are shown below in Supplementary Table 3.

**Supplementary Table 3**. Results of the Cox Proportional Hazards test evaluating survival in hypertensive rats

| Factor | P-value | Hazard Ratio (95% CI) |
| --- | --- | --- |
| VNS Therapy | 0.008 | 0.076 (0.011 ± 0.52) |
| Baseline MAP | 0.16 | 3.61 (0.60 ± 21.75) |
| Baseline DBP | 0.99 | 0.99 (0.50 ± 1.97) |
| Baseline SBP | 0.11 | 0.29 (0.064 ± 1.32) |
| Baseline HR | 0.78 | 1.00 (0.97 ± 1.04) |
| Baseline PP | 0.09 | 2.24 (0.89 ± 5.67) |

**
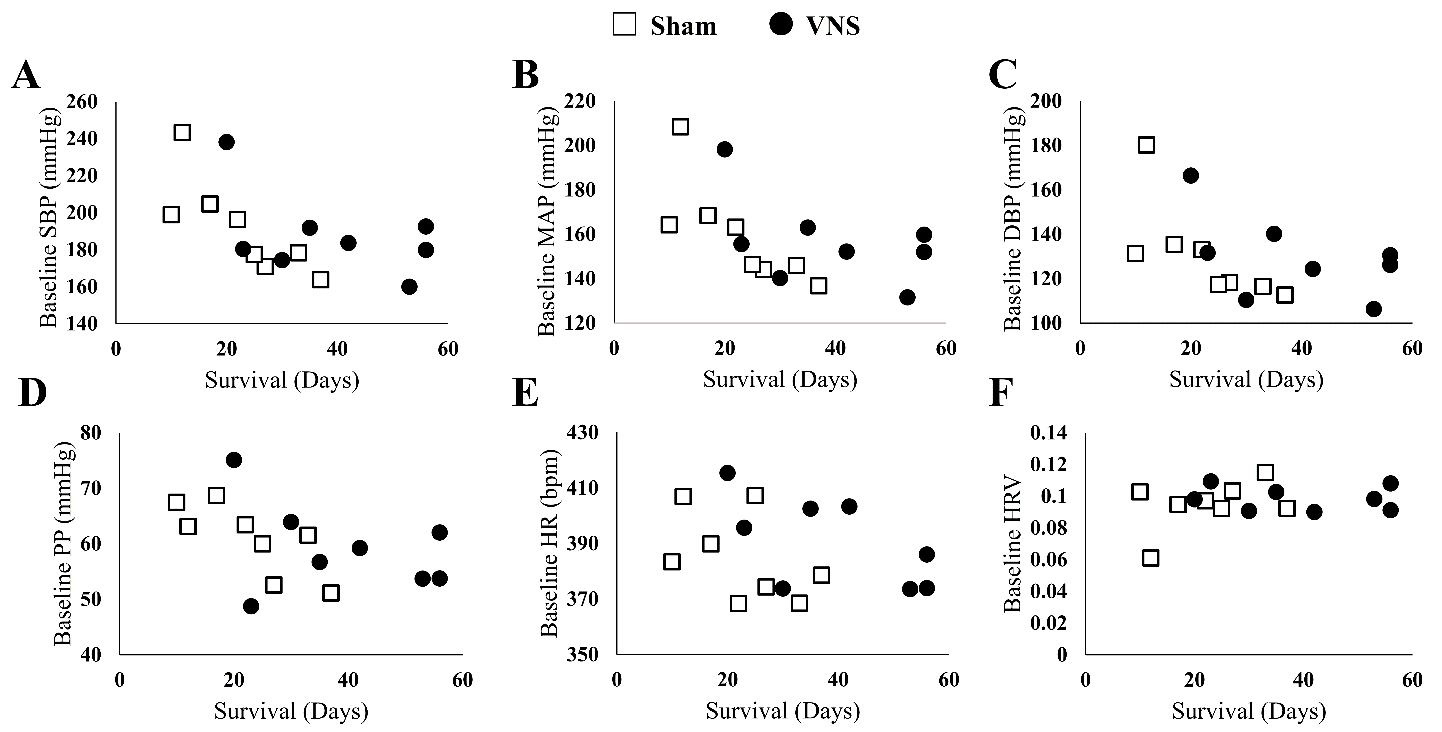
**

**Supplementary Figure 1.** Baseline characteristics of Sham (n=8) and VNS (n=9) rats versus event-free survival times. Baseline SBP (A), MAP (B), DBP (C), PP (D), HR (E), and HRV (F) show no significant differences between groups demonstrating no significant influence on the differences observed in event-free survival times in Sham and VNS rats.

## Impact of VNS on event-free survival

The summary of endpoints for the hypertensive rats are shown below in Supplementary Table 3. The most prevalent endpoint for this study for VNS and Sham rats was hypertension-related adverse event, specifically stroke. However, two rats reached death as their endpoint, both of which occurred during the night time (between 12am-4am). Similarly, for those rats that had a hypertension-related adverse event, the majority of events also occurred during the night time intervals of the study while lights in the room are turned off (7pm-7am). For those rats (2 VNS) that experienced spontaneous death, the tissues were not included for an analysis, as they were not collected for histology and gross morphology analysis. For the remaining rats (8 Sham; 6 VNS), at the time the endpoint was, tissues were all collected in the manner described in the methods.

**Supplementary Table 4**. Summary of study endpoints in Sham and VNS rats

| **Treatment Group** | **Survival (Days)** | **Endpoint** |
| --- | --- | --- |
| Sham | 10 | Hypertension-related complication: retinal hematoma |
|  | 12 | Hypertension-related complication: blind, balance deficiencies. |
|  | 17 | Hypertension-related complication: stroke, loss of limb function |
|  | 22 | Hypertension-related complication: stroke |
|  | 25 | Hypertension-related complication: Blind, retinal bleeding |
|  | 27 | Hypertension-related complication: stroke, loss of limb function and partial blindness |
|  | 33 | Hypertension-related complication: stroke, loss of limb function and partial blindness |
|  | 37 | Hypertension-related complication: blindness and retinal hemorrhage |
|  |  |  |
| VNS | 20 | Hypertension-related complication: stroke |
|  | 23 | Died* |
|  | 30 | Died* |
|  | 31 | VNS lead stopped pacing during W11  Rat survival data point was excluded from analysis |
|  | 35 | Hypertension-related complication: stroke, loss of limb function |
|  | 42 | Hypertension-related complication: stroke |
|  | 53 | Hypertension-related complication: stroke, loss of limb function |
|  | 56 | Survived |
|  | 56 | Survived |

* Rats died over night, and tissues were not collected.

## Impact of VNS on acute cardiovascular and hemodynamic response

At Week 12, the remaining VNS rats were evaluated for their acute cardiovascular and hemodynamic response to stimulation (Figure S2). No significant differences were observed in SBP, BPV, HR, or contractility values. However, HRV demonstrated a similar response to Week 9 with a significant acute increase in HRV during “VNS On” in comparison to the “Post 2” interval.

**
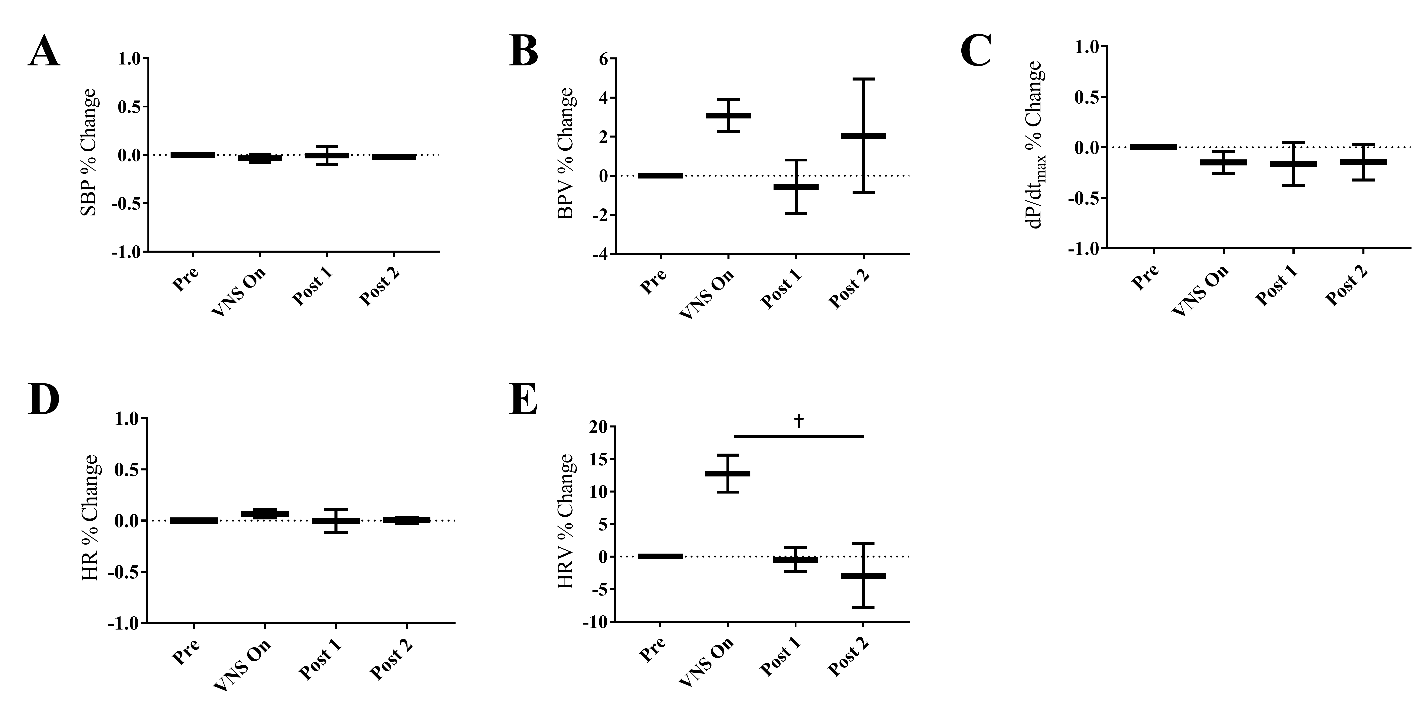
**

**Supplementary Figure 2.** Acute hemodynamic effects of VNS after 6 weeks of therapy. Acute SBP (A), BPV (B), contractility (C), HR (D), and HRV (E) responses during VNS therapy in hypertensive rats for “Pre”, “VNS On”, “Post 1”, and “Post 2” intervals. * indicates statistical significance between intervals.

## Impact of VNS on disease progression

Longitudinal analyses were performed on additional BP measures including MAP, DBP, and PP, which are shown in Figures S3-S5. The disease progression of MAP, shown in Figure S3 panel A and B for *Day_12h_* and *Night_12h_* intervals respectively, shows an attenuation in ΔMAP for VNS rats in comparison to Sham rats. This attenuation, quantified by the slope of the linear regression, is significant during the *Night_12h_* interval (Sham: 13.1 ± 1.6 mmHg/week; VNS: 8.5 ± 1.1 mmHg/week; P < 0.05) but not the *Day_12h_* interval (Sham: 13.7 ± 1.7 mmHg/week; VNS: 9.2 ± 1.1 mmHg/week; P = 0.1029). Circadian rhythm analysis at Week 6 and Week 9 are shown in panels C and D. There are no differences in circadian characteristics at baseline. However, after three weeks of therapy, there is a significant separation in the MESOR value, with Sham rats having a significantly higher MAP MESOR than the VNS rats.

The progression of DBP from Week 6 to Week 9 during *Dayt_12h_* and *Night_12h_* are shown in Figure S4, panel A and B. Although there is a separation in the ΔDBP, it is not significantly different between Sham and VNS rats during either the *Day_12h_* or *Night_12h_* interval. The results of the circadian rhythm analysis are shown in panels C and D for Week 6 and Week 9 respectively. Again, there were no significantly differences in the circadian rhythm characteristics at baseline, and after three weeks of therapy there was a significant difference in the MESOR values between the Sham and VNS rats. The Sham rats had a significantly larger DBP MESOR value.

Figure S5 shows the progression of PP in the Sham and VNS rats over the first three weeks of therapy. ΔPP showed similar results, with no significant differences in ΔPP between groups during either the *Day_12h_* or *Night_12h_* interval. In addition, the circadian rhythm analysis produced similar results with no differences observed at Week 6 and a significant increase in the PP MESOR value for Sham rats compared to VNS rats.

**
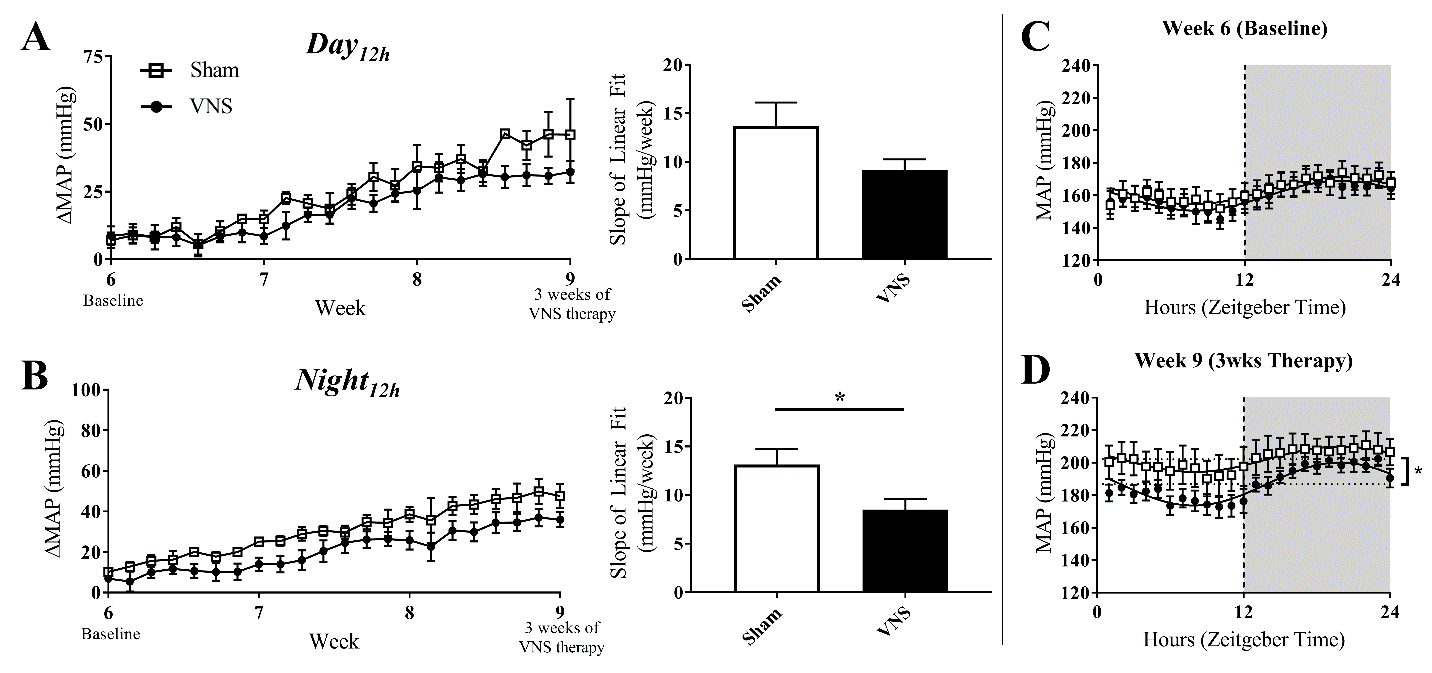
**

**Supplementary Figure 3.** Disease progression of mean arterial pressure (MAP). Relative change in MAP between Sham and VNS groups during the (A) *Day_12h_* interval and the (B) *Night_12h_* interval. Linear slopes were fit individually to each rat and compared between groups for the *Day_12h_* and *Night_12h_* intervals. (C-D) Circadian rhythm analysis of MAP for Sham and VNS rats at Week 6 (Sham: n=8; VNS: n=9), baseline, and at Week 9 (Sham: n=5; VNS: n=8), after three weeks of therapy. * indicates statistical significance between Sham and VNS rats.


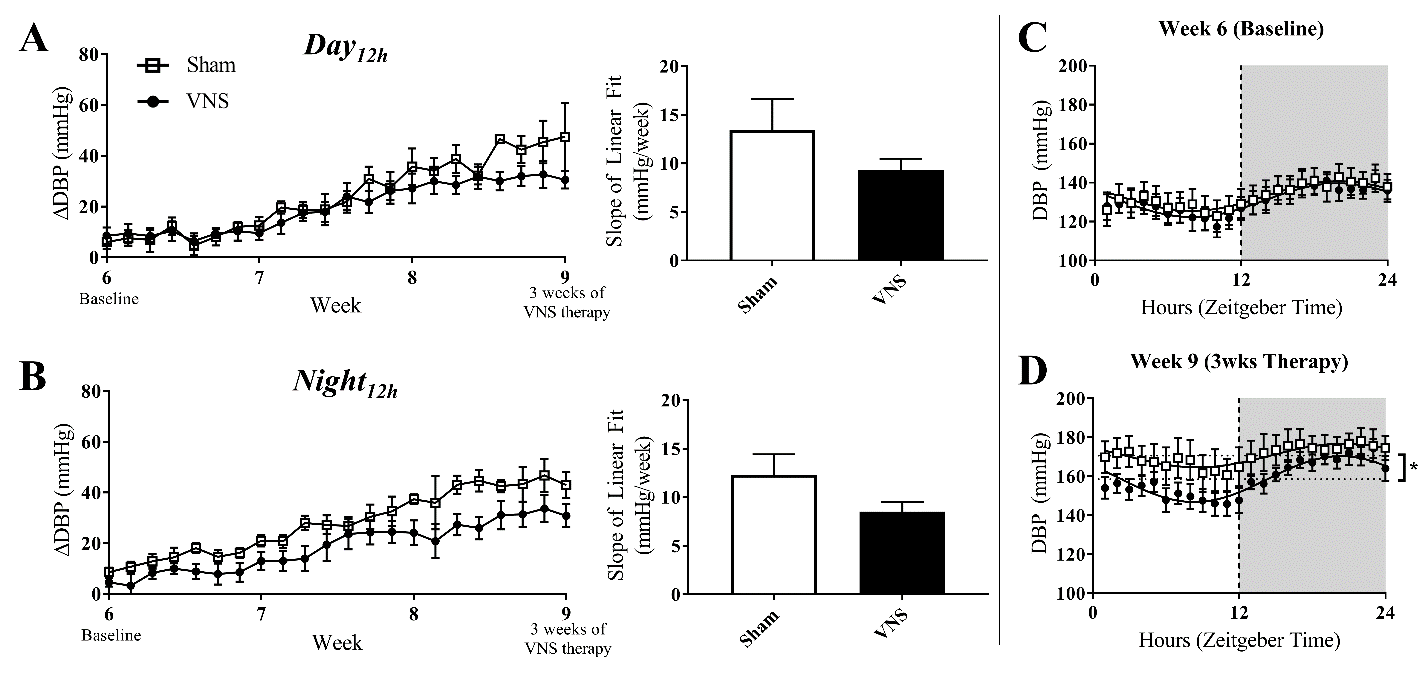


**Supplementary Figure 4.** Disease progression of diastolic blood pressure (DBP). Relative change in DBP between Sham and VNS groups during the (A) *Day_12h_* interval and the (B) *Night_12h_* interval. Linear slopes were fit individually to each rat and compared between groups for the *Day_12h_* and *Night_12h_* intervals. (C-D) Circadian rhythm analysis of DBP for Sham and VNS rats at Week 6 (Sham: n=8; VNS: n=9), baseline, and at Week 9 (Sham: n=5; VNS: n=8), after three weeks of therapy. * indicates statistical significance between Sham and VNS rats.


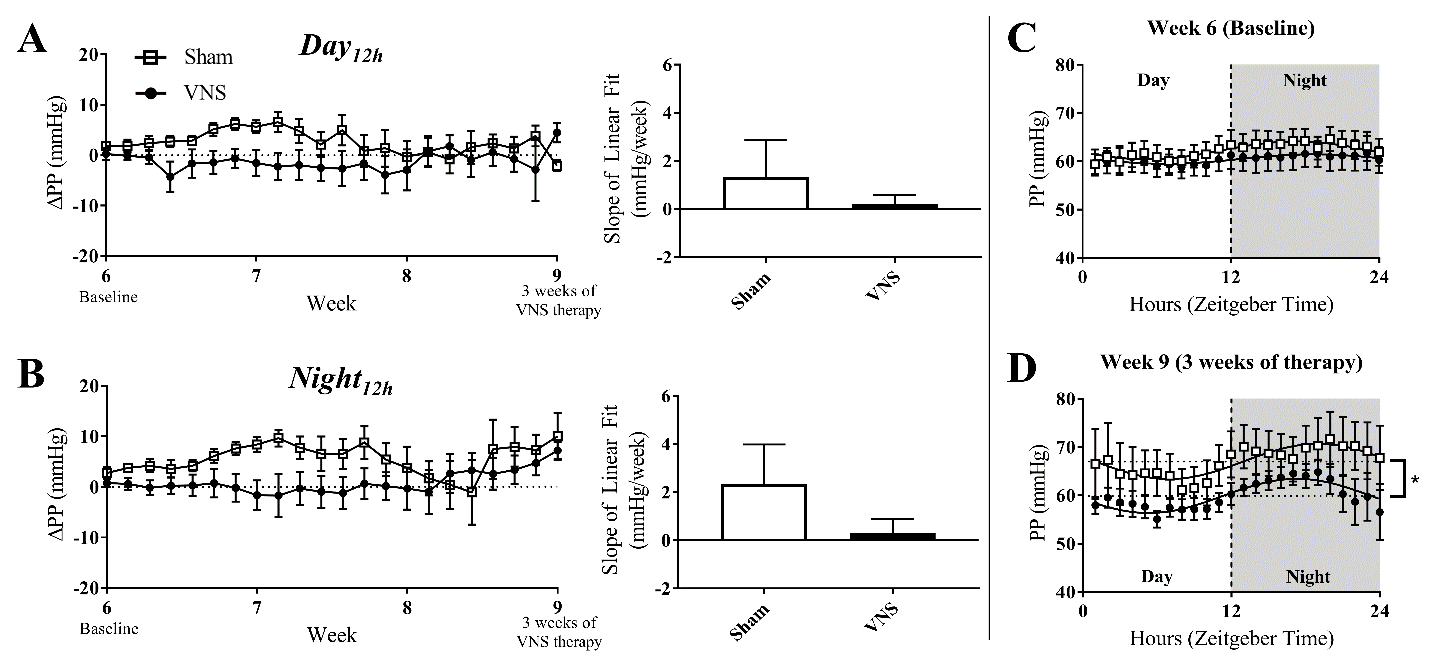


**Supplementary Figure 5.** Disease progression of pulse pressure (PP). Relative change in PP between Sham and VNS groups during the (A) *Day_12h_* interval and the (B) *Night_12h_* interval. Linear slopes were fit individually to each rat and compared between groups for the *Day_12h_* and *Night_12h_* intervals. (C-D) Circadian rhythm analysis of PP for Sham and VNS rats at Week 6 (Sham: n=8; VNS: n=9), baseline, and at Week 9 (Sham: n=5; VNS: n=8), after three weeks of therapy. * indicates statistical significance between Sham and VNS rats.

Throughout the course of the study, weight was monitored between groups and are shown below in Supplementary Figure 6. There were no significant differences between weights in the two groups from Week 6-8. However, at Week 9, VNS rats had a significantly larger weight. Although if you remove the two Sham rats that were near their endpoint (within 1 week) the data at Week 9 shows no significance. As rats neared their endpoints, they began to trend downward in weight measures.


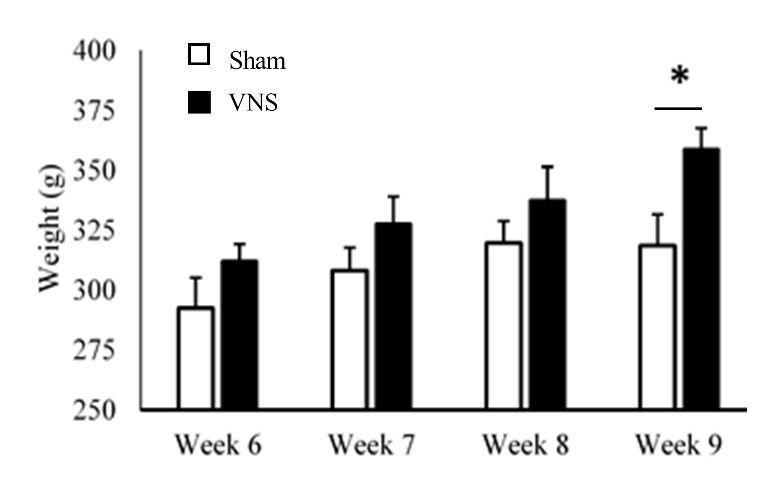


Supplementary Figure 6. Weight measurements for Sham and VNS rats from Week 6 (Sham: n=8; VNS: n=9), baseline, and at Week 9 (Sham: n=5; VNS: n=8),

## Structural cardiac effects


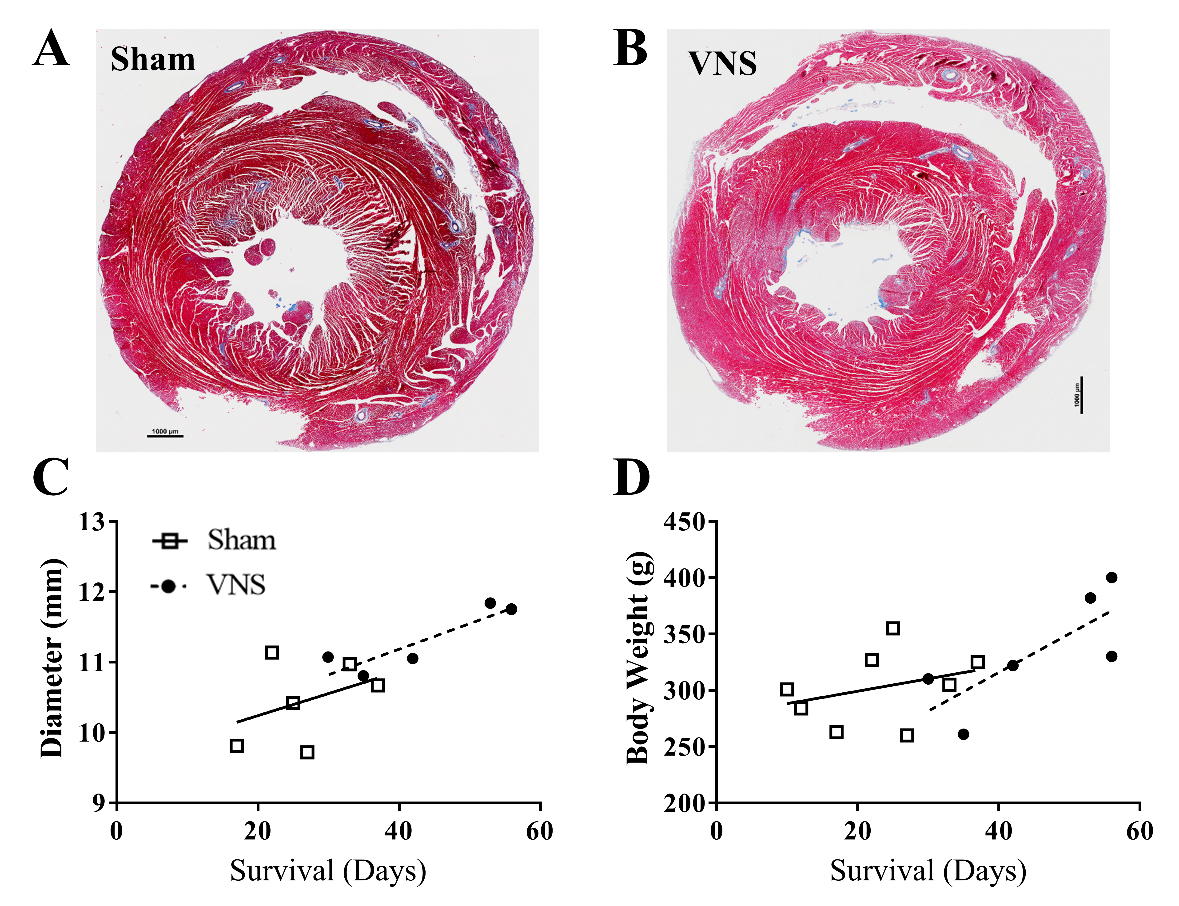


**Supplementary Figure 7.** Examples of the cardiac cross sections from the Sham (A) and VNS (B) hearts stained with Masson’s Trichrome. The cardiac fibrosis can be seen in blue around the vessels and within the interstitial space. (C) Cross section diameter (Sham: n=6; VNS: n=6) and (D) body weight as a function of survival times with corresponding linear regressions for Sham (n=8) and VNS rats (n=6).

**Supplementary Table 5**. Summary of structural parameters for Sham and VNS rats

| **Treatment Group** | **Survival (days)** | **HW (g)** | **TL (cm)** | **Diameter (um)** | **RV Thickness (um)** | **LV Thickness (um)** | **Septum Thickness (um)** | **% Fibrosis** |
| --- | --- | --- | --- | --- | --- | --- | --- | --- |
| **Sham** | 10 | 1.265 | 4.1 | * | 1225 | 3137 | 2753 | 2.69 |
|  | 12 | 1.838 | 4.1 | * | 902 | 3084 | 2266 | 6.49 |
|  | 17 | 1.648 | 4.17 | 9815 | 881 | 2899 | 2318 | 4.71 |
|  | 22 | 1.561 | 4.21 | 11139 | 1325 | 2970 | 3091 | 4.87 |
|  | 25 | 1.517 | 4.23 | 10423 | 1108 | 3129 | 2608 | 3.24 |
|  | 27 | 1.198 | 3.91 | 9721 | 1186 | 3354 | 2597 | 2.33 |
|  | 33 | 1.283 | 4.23 | 10971 | 1069 | 3524 | 2550 | 3.57 |
|  | 37 | 1.374 | 4.15 | 10670 | 1095 | 2970 | 2828 | 2.44 |
| **VNS** | 30 | 1.457 | 4.47 | 11071 | 1407 | 3375 | 2941 | 2.58 |
|  | 35 | 1.534 | 4.23 | 10805 | 926 | 2523 | 2440 | 4.98 |
|  | 42 | 1.392 | 4.26 | 11053 | 1243 | 3334 | 3081 | 2.43 |
|  | 53 | 1.590 | 4.25 | 11837 | 962 | 2709 | 2221 | 2.81 |
|  | 56 | 1.969 | 4.32 | 11757 | 1056 | 2751 | 3041 | 2.78 |
|  | 56 | 1.897 | 4.48 | 11750 | 1096 | 3368 | 3477 | 5.08 |

*indicates that a diameter measure could not be made from histology cross-sections

# References

Shinlapawittayatorn, K., Chinda, K., Palee, S., Surinkaew, S., Kumfu, S., Kumphune, S., . . . Chattipakorn, N. (2014). Vagus nerve stimulation initiated late during ischemia, but not reperfusion, exerts cardioprotection via amelioration of cardiac mitochondrial dysfunction. *Heart Rhythm, 11*(12), 2278-2287. doi:10.1016/j.hrthm.2014.08.001

Veitenheimer, B., & Osborn, J. W. (2011). Role of spinal V1a receptors in regulation of arterial pressure during acute and chronic osmotic stress. *American Journal of Physiology-Regulatory Integrative and Comparative Physiology, 300*(2), R460-R469. doi:10.1152/ajpregu.00371.2010

Xie, X., Lee, S. W., Johnson, C., Ippolito, J., KenKnight, B. H., Tolkacheva, E. G., & Ieee. (2014). *Intermittent Vagal Nerve Stimulation Alters the Electrophysiological Properties of Atrium in the Myocardial Infarction Rat Model.* Paper presented at the 36th Annual International Conference of the IEEE-Engineering-in-Medicine-and-Biology-Society (EMBC), Chicago, IL.
